# Suspicion of mitochondrial disease remains frequently unconfirmed after whole exome sequencing

**DOI:** 10.1016/j.ymgmr.2018.07.005

**Published:** 2018-07-27

**Authors:** Josef Finsterer, Sinda Zarrouk-Mahjoub

**Affiliations:** a Krankenanstalt Rudolfstiftung, Vienna, Austria; b University of Tunis El Manar and Genomics Platform, Pasteur Institute of Tunis, Tunisia

**Keywords:** Mitochondrial, mtDNA depletion, Whole exome sequencing, Phenotype, Genotype, multisystem disease, lactic acidosis

Letter to the Editor,

We read with interest the article by Pausepp et al. about 28 patients with suspected mitochondrial disorder (MID) who retrospectively (*n* = 17) or prospectively (*n* = 11) had undergone whole exome sequencing (WES) [1]. We have the following comments/concerns.

The authors mention that only in 4/28 patients a MID was genetically confirmed [1]. However, in one further patient an *ATP6* variant was detected and in another patient mtDNA depletion was detected on muscle biopsy [1]. Furthermore, the *PDHA1* carrier should be regarded as a MID [2]. Why were these three patients not included in the MID-group?

Only in 5 of 28 patients did the authors detect a mutated gene associated with a MID and in 1 patient a possible mitochondrial depletion syndrome (MDS) was diagnosed? Were any of the genes associated with MDS screened for a mutation [3]? In how many of the 6 patients with a MID was the family history positive for the disease? In the patient carrying the *ATP6* mutation, no heteroplasmy rates are provided. In which tissue were heteroplasmy rates determined and was the degree of heteroplasmy compatible with the phenotype? Did the mother of this patient also carry the mutation?

Spinal muscular atrophy (SMA) should not be diagnosed upon the muscle biopsy findings. Did the patient with “typical” histological features of SMA also carry a deletion/duplication of the *SMN1* gene?

Muscle hypotonia was reported in 11 patients [1]. Was hypotonia attributed to a central cause or due to involvement of the peripheral nerves or the muscle? Only one patient had neuropathy but 16 had muscle weakness [1].

Overall, the study may profit from inclusion of the family history, from provision of heteroplasmy rates for the *ATP6* variant, and from stressing that clinical assessment with or without scores may falsely suggest MID in a significant number (*n* = 21) of patients.

## Conflict of interest

There are no conflicts of interest.

## Funding

No funding was received.

## Author contribution

JF: design, literature search, discussion, first draft, SZM:literature search, critical review.
